# Perfluorocarbon nanoemulsions in drug delivery: design, development, and manufacturing

**DOI:** 10.7150/thno.103820

**Published:** 2025-02-10

**Authors:** Riddhi Vichare, Jelena Janjic

**Affiliations:** School of Pharmacy, Graduate School of Pharmaceutical Sciences, Duquesne University, Pittsburgh, PA 15282, USA.

**Keywords:** Perfluorocarbons, Nanoemulsions, Drug delivery, Theranostic, Targeted drug delivery, Manufacturing, Quality by Design

## Abstract

Perfluorocarbons (PFCs) are formulated into kinetically stable nanoemulsions (NEs) having a droplet diameter less than 500 nm for biomedical applications. PFC NEs are developed for various applications, such as ^19^F magnetic resonance imaging tracers, ultrasound contrast agents, and oxygen carriers. They are an attractive platform for theranostic development, as PFC NEs can be designed to deliver a multitude of therapeutics, from small molecules to biologics. Although many reviews have been published on designing PFC NEs as imaging agents or oxygen carriers, there is no comprehensive review of the formulation strategies and manufacturing of PFC NEs as drug delivery platform. From a “formulator's perspective,” the presented review addresses this gap as it describes the selection of formulation components such as the PFCs, surfactants, and hydrocarbon oils and provides an overview of manufacturing techniques. In this review, we will also identify potential shortcomings in the published literature on the manufacturing of PFC NEs. We will discuss the need for the implementing Quality by Design (QbD) approach in the manufacturing of PFC NEs to achieve scalability and necessary quality control for supporting both preclinical and clinical applications. Finally, we will review different surface-conjugation strategies for developing targeted PFC NEs. Overall, this article aims to increase understanding of PFC NEs designed for delivering drugs, including both small molecules and biologics.

## 1. Introduction

### 1.1 Brief Overview of Perfluorocarbon Nanoemulsions (PFC NEs)

Perfluorocarbons **(PFCs)** were initially synthesized as inert solvents for separating uranium isotopes 1. It was not until 1966, when Clark and Gollan, in their 'liquid breathing' experiment, delineated the use of oxygen-saturated PFC liquids as an alternative respiratory medium 2. PFCs are colorless and odorless synthetic compounds in which all the hydrogen atoms on a saturated linear or cyclic carbon backbone are substituted with fluorine atoms, with a few exceptions where heteroatoms are present in the molecular structure 3. PFCs are chemically inert due to their electronic structure and the spatial arrangement of fluorine atoms on the C-C backbone. Fluorine substitution changes the physicochemical properties of the hydrocarbon bond due to its high electronegativity, high ionization potential, and low polarizability compared to the hydrogen atom. The C-F bond is a stronger covalent single bond compared to the C-H bond, requiring roughly 530 kJmol^-1^ of energy to break the bond. Additionally, fluorine substitutions force the C-C backbone to adopt a helical configuration, deviating from the planar zigzag configuration observed in the presence of hydrogen atoms 3, 4. The large trans/gauche conversion energy barrier (4.6 kJmol^-1^) imparts rigidity to the structure, sterically shielding the C-C bonds from chemical attacks 5. Early on, the scientific community recognized the need to emulsify PFCs for clinical applications involving parenteral administration to prevent fatal vascular embolism 2, 6. In 1967, Solviter was the first to report the emulsification of PFCs in water using bovine serum albumin through sonication (droplet diameter: 2-3
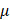
m) for perfusing isolated rat brains 7. Around the 1980s, the HIV-virus-contaminated donor blood epidemic further intensified research on PFCs as artificial blood substitutes owing to their high oxygen-carrying capacity 8. Although termed blood substitutes, PFC emulsions did not perform all the functions of blood, like coagulation and nutrient transport. In 1989, Fluosol-DA^®^ (droplet diameter < 200 nm) became the first FDA-approved oxygen-carrying PFC emulsion for perfusing coronary arteries, which was manufactured using the high-pressure homogenization technique 9. Unfortunately, Fluosol-DA^®^ was withdrawn from the market mainly due to its poor stability and low oxygen-carrying capacity. In parallel, a paper discussing PFCs as tracer agents for ^19^F magnetic resonance imaging **(MRI)** was published 10. The ^19^F atom has a negligible biological abundance, and therefore fluorinated materials can be non-invasively detected *in vivo* using ^19^F MRI without background 11. Indeed, the late 20^th^ century was a “golden period” for PFCs, officially marking their entry into the biomedical field.

During the same timeline, emulsification technology extended from the food industry to the pharmaceutical sector. Consequently, a metric prefix terminology, nanoemulsions **(NEs)**, was introduced in 1996 for describing droplets having a diameter in the nanometer range 12, 13. NEs are kinetically stable colloidal dispersions of two immiscible liquids (oil and water), where a high oil fraction is stabilized with a low surfactant concentration (5-10% w/v). This is distinct from thermodynamically stable microemulsions, which are formulated at a higher surfactant-to-oil ratio **(SOR)** 14. There is no consensus within the field over the upper limit of droplet size considered acceptable for NEs. The droplet size of NEs, particularly PFC NEs, formulated for clinical applications ranges between 100 nm and 500 nm 15-19. Through continued research efforts, PFC NEs are being designed and formulated to serve as ^19^F MRI tracers, ultrasound contrast agents, *ex vivo* cell labeling agents, probes for intracellular pH measurements, and as oxygen carriers in regenerative medicine and oncology to reverse tissue hypoxia, which are comprehensively covered elsewhere 20-26.

Recently, research interest has shifted towards designing PFC NEs as drug delivery platform, with their imaging or oxygen-carrying capabilities serving as added functionalities. This interest is primarily because of the biologically inert nature of PFCs. Following intravenous (*i.v*) administration, PFC NE droplets are removed from the bloodstream by the reticuloendothelial system (RES), with the liver and spleen playing a major role in this process. In RES organs, PFC NEs are first demulsified, and then PFC droplets are transported across cell membranes to blood vessels at a rate contingent on the lipophilicity of the PFC. Subsequently, PFC droplets are taken up by circulating lipoproteins and transported to the lung, where they are eventually excreted unmetabolized through exhalation 27, 28. Typically, PFC NEs for oxygen delivery (Oxygent^TM^ and Oxycyte^®^) are formulated at a higher PFC concentration (~60% w/v) 29, 30. Whereas for formulating PFC NEs for successful drug delivery and cell labeling applications, PFC concentrations up to 30% w/v are reported. Unlike the existing literature reviews, which primarily focus on the diagnostic or oxygen delivery aspect of PFC NEs, this review will summarize the selection of different components used while formulating PFC NEs as delivery vehicles for small molecules and biologics. We will discuss two distinct processing techniques, microfluidization and ultrasonication, commonly reported for manufacturing PFC NEs. Furthermore, we will enlist a few surface conjugation strategies used for developing targeted PFC NEs. A differentiating factor of this review is that we will identify key problems in the published literature on PFC NE manufacturing and introduce application of Quality by Design (QbD) for developing PFC NEs for drug delivery.

### 1.2 Types of Perfluorocarbon Nanoemulsions

**Figure 1:**
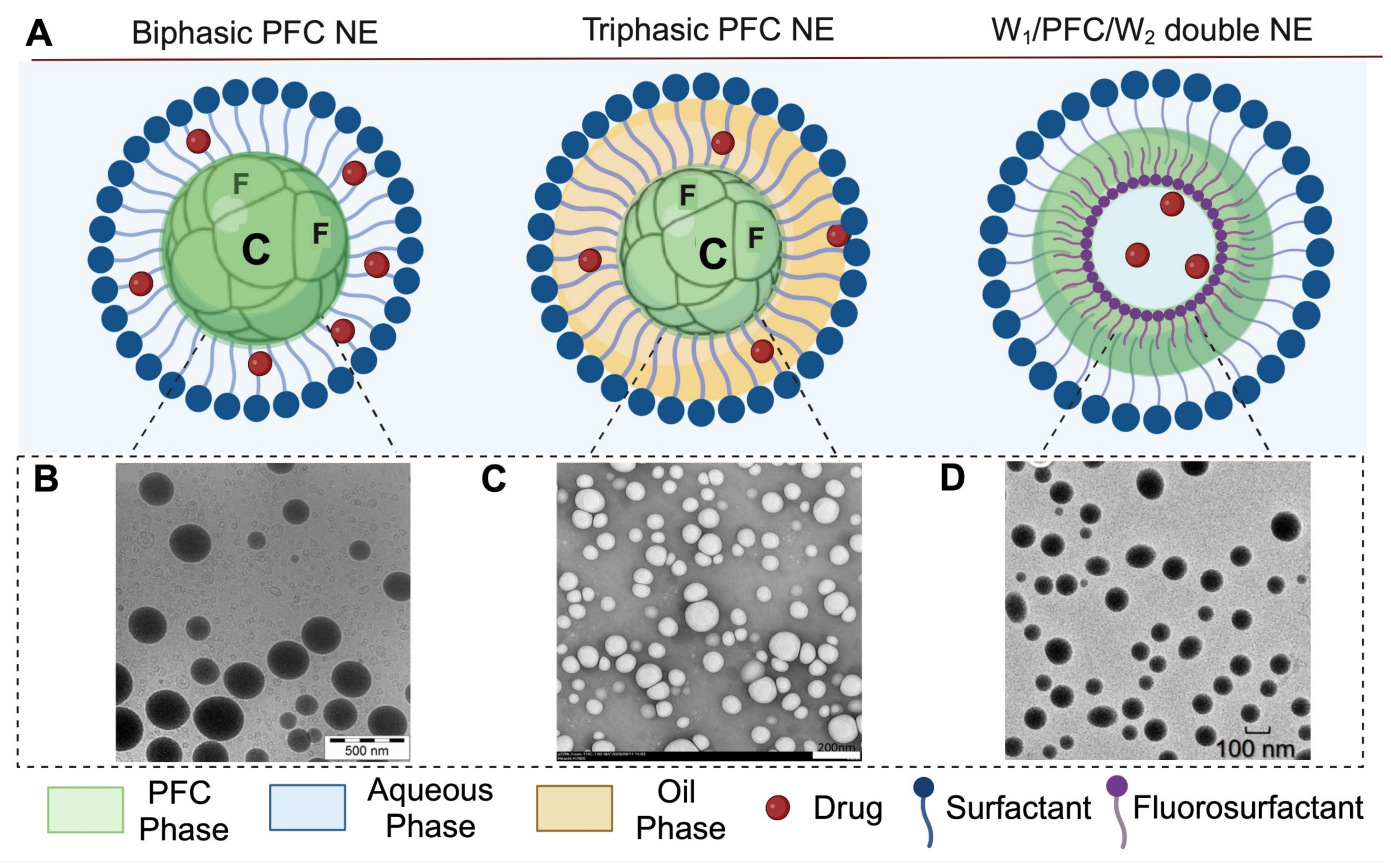
**Compositional differences between PFC NEs. A.** Illustrates the types of PFC NEs used for drug delivery applications. Created with BioRender.com. **B.** The image of Cryo-Transmission Electron Microscopy (TEM) for heat sterilized PFOB NE emulsified with phospholipids was observed using a Leo 912 Ω-mega, Carl Zeiss instrument operated at 120 kV. Figure adapted from 31 under the Creative Commons Attribution License (CC BY 4.0), PLOS One. **C.** Cryo-TEM image for PFC NE with PFOB, Tributyl O-acetyl Citrate oil, and fluorinated surfactant was observed using a MET Hitachi instrument operated at 80 kV. Figure adapted from 34 under the Creative Commons Attribution License (CC BY 4.0), MDPI. **D.** Cryo-TEM image of W_1_/PFOB/W_2_ double nanoemulsions stabilized by polyethoxylated fluorosurfactant and carboxylic Pluronic F-68 copolymer was observed using a JEM-1400. Figure adapted from 37 under the Creative Commons Attribution License (CC BY 4.0), MDPI.

Perfluorocarbon nanoemulsions for drug delivery applications can be formulated as biphasic PFC NEs, which consist of PFC as the dispersed phase, water as the continuous phase, and surfactant to stabilize the interface 31. Exceptions to this commonly reported type are triphasic PFC NEs and W_1_/PFC/W_2_ double nanoemulsions. In triphasic PFC NEs, hydrocarbon oil is incorporated as an additional dispersed phase along with PFC, water, and surfactants 32-34. In the presence of oil, PFCs form a third “fluorous” phase owing to their low propensity to participate in induced dipole-induced dipole interactions with the hydrocarbon oil and induced dipole-dipole interactions with the water 3. In order to formulate W_1_/PFC/W_2_ double nanoemulsions, Lee Y-H and coworkers used a combination of surfactants and followed the two-step emulsification technique. In the first step, a water-in-PFC (W_1_/PFC) emulsion was developed by emulsifying PFC in the water phase using polyethoxylated fluorosurfactant. This primary emulsion was later added to a solution of triblock copolymer and subjected to sonication to formulate a W_1_/PFC/W_2_ double nanoemulsion 35-37. Compositional differences between the three types of PFC NEs are depicted in **Figure 1A-D**.

## 2. Design and Development of PFC NEs as a Drug Delivery Platform

Perfluorocarbon nanoemulsions are a versatile drug delivery platform, reflected by their ability to deliver different therapeutic payloads ranging from small molecules 16, 17, 35, 37-43 to biologics such as small interfering RNAs **(siRNAs)** 18, 44, 45, proteins 19, 46, peptides and enzymes 47-50, and oligonucleotides 51 **(Table 1)**.

**Figure 2:**
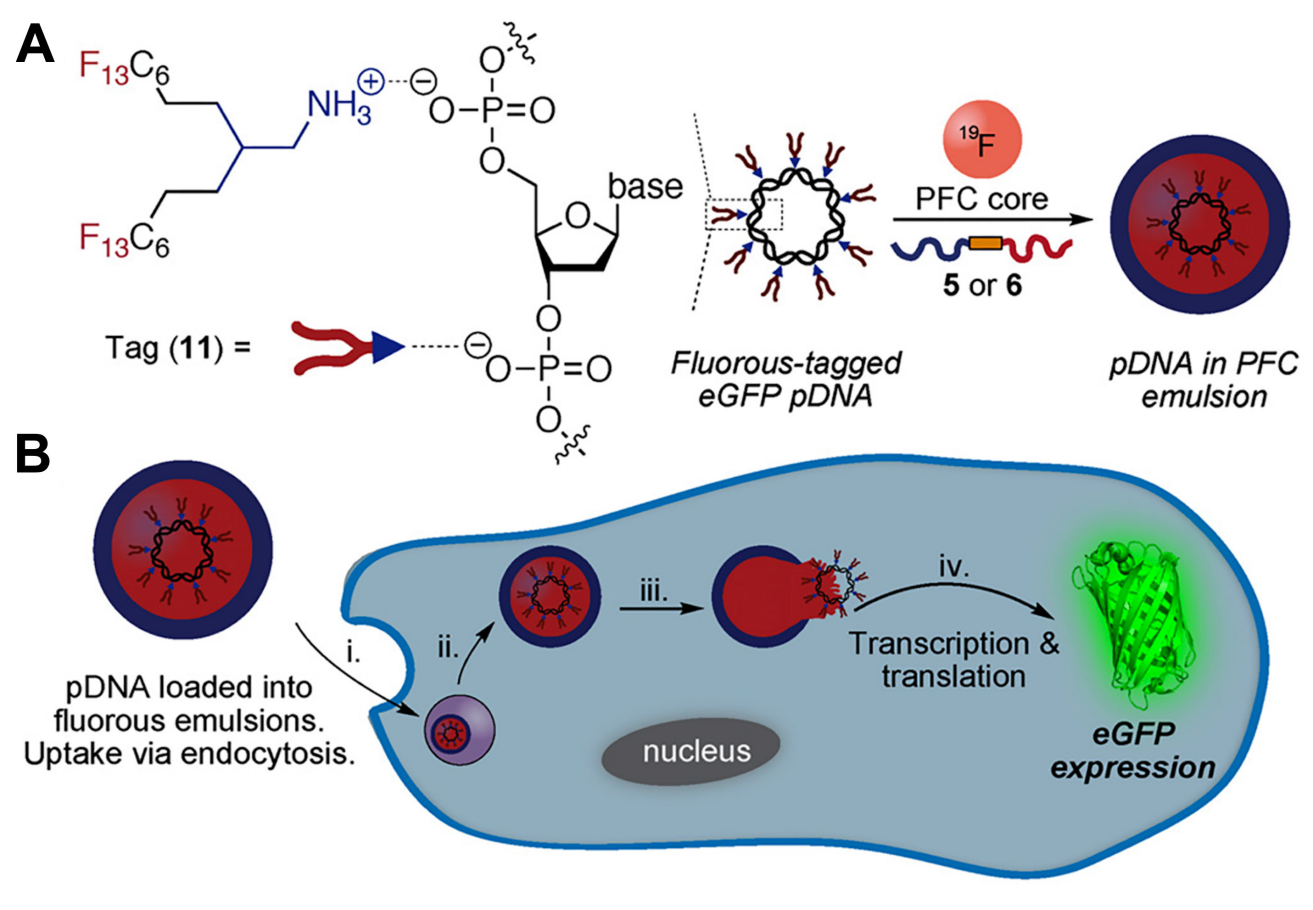
** Strategy for loading pDNA into PFC core**. **A.** Fluorous amine tag complexes with eGFP pDNA (model pDNA) to facilitate its solubilization in the PFC liquid. In the next step, the fluorous-tagged eGFP pDNA was sonicated in the presence of a reduction-sensitive surfactant, PFC, and water to form eGFP-loaded PFC NE. **B.** Schematic of *in cellulo* eGFP pDNA delivery. The cell uptake of eGFP pDNA-loaded PFC NE *via* endocytosis (i). This is followed by endosomal escape in cytosol (ii), and release of eGFP pDNA due to reduction of disulfide-linked surfactant in presence of cytosol glutathione (iii). Nuclear entry and expression of eGFP (iv). Reprinted with permission from 51. Copyright (2021) from John Wiley and Sons, Inc.

In biphasic PFC NEs, poorly soluble drugs or biologics are incorporated into the surfactant layer. In triphasic PFC NEs, small molecules are loaded in the oil phase. As NEs are formulated using low surfactant concentrations, the amount of therapeutic drug that can be loaded into the surfactant layer is limited. By incorporating an oil phase, it is feasible to improve the capacity for loading poorly soluble drugs or lipophilic dyes 33. In recent research, an alternative strategy has been investigated where the therapeutic payloads are dispersed within the PFC core of biphasic PFC NEs. This approach is particularly applied for delivering macromolecules, as the fluorous PFC core can efficiently shield the biological cargo from an external degradative environment 19, 51. Sloand and coworkers 19 screened a small library of fluoroalkanes, carboxylic acid fluoroalkanes, and fluorobenzenes to select optimal fluorous tags (FTags). Mechanistically, FTags can “fluorous mask” the protein of interest to facilitate its dispersion in the PFC core. This is in contrast of loading the macromolecule in the surfactant layer. The research group demonstrated that the selected FTag (perfluorononanoic acid) noncovalently complexes with the model protein via hydrogen bonds that are formed between the carboxylic end of the FTag and the polar side chains of the protein. Importantly, the chemical modification with the FTag did not compromise the native conformational state and retained the biological function of the protein. In another study based on the same concept, Estabrook and coworkers demonstrated that cationic tags (ammonium with two C_6_F_13_ chains) can associate with the anionic backbone of pDNA through electrostatic interactions, which allows the fluorous-tagged pDNA to be loaded into the PFC core of the NE. The fluorous-tagged eGFP pDNA was loaded in PFC NE and was successfully delivered to promote *in cellulo* eGFP expression **(Figure 2A and B)** 51.

The success of PFC NEs as a drug delivery platform relies on the selection of formulation components like PFC, surfactant, and hydrocarbon oil. As described below, through this careful selection, formulators can design PFC NEs that not only deliver therapeutic payloads but also offer additional benefits such as diagnostic capability or provide spatiotemporal control over drug release.

### 2.1 Selection of PFCs: Building Blocks of PFC NEs

**Figure 3:**
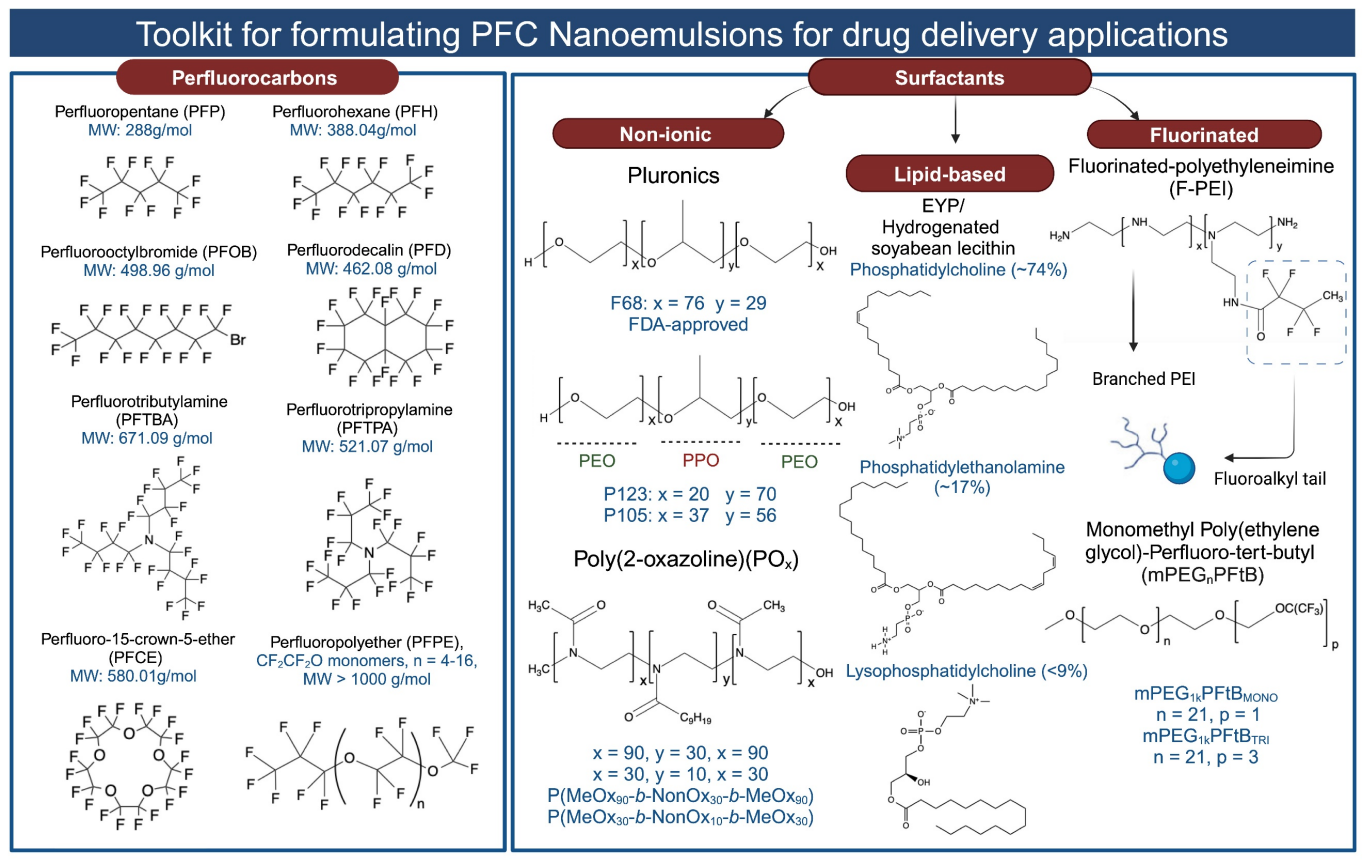
Structures of the PFCs and surfactants commonly used for formulating PFC NEs for drug delivery. Created with Biorender.com. All the chemical structures were drawn using ChemDraw 21.0.0.

PFCs used for drug delivery applications can be broadly categorized into two groups: (1) Perfluorinated compounds with a molecular weight less than 1000 g/mol. This group includes compounds like perfluorooctyl bromide **(PFOB)**, perfluoro-15-crown-5-ether **(PFCE)**, perfluorodecalin **(PFD)**, perfluorotributyl amine (PFTBA), and others. (2) Perfluorinated polymers with a molecular weight greater than 1000 g/mol. An example in this category is perfluoropolyether **(PFPE)** (for all chemical structures and molecular weights, refer to **Figure 3**). When selecting PFCs for drug delivery applications, organ clearance, which is inversely proportional to the molecular weight of the PFC, has been given precedence primarily to avoid long-term organ retention. Although for most PFCs, molecular weight is a reliable predictor of organ clearance, there are a few exceptions to this rule. For example, PFOB shows faster organ clearance (~4 days) compared to perfluorotripropylamine (~65 days), despite a comparable molecular weight 4. This is attributed to the presence of a polarizable bromine atom at the terminal position of PFOB, which increases its molecular lipophilicity. As a result, circulating lipoproteins can easily take up PFOB and transport it for pulmonary removal 27, 52. Bérard *et al.* 34 recently investigated the *in vivo* biodistribution of PFOB NE in major organs at different time points post *i.v* injection. The authors' data corroborated the value reported in the literature, as PFOB was completely excreted from major organs as early as 3 days without any signs of organ toxicity. PFOB is commonly selected by researchers for developing drug-delivering PFC NEs 18, 42, 49 based on its favorable organ clearance time and proven safety up to 1.35 g of PFOB dose/kg body weight in humans 29.

A molecularly symmetric PFCE is selected when the goal is to develop theranostic PFC NEs 17, 38, 53, 54. Theranostic nanomedicines represent a recent advancement in the field of drug delivery, designed to simultaneously perform therapeutic and diagnostic functions. The capability to image the therapeutic during or after administration presents a prospect for developing personalized medicines in the future 55. Despite the favorable organ clearance time, PFOB is not ideal for imaging purposes. The ^19^F NMR spectra of PFOB consist of eight resonance peaks. Therefore, special frequency-selective MRI pulse sequences and extended scan times are required to improve the spatial resolution of images by minimizing chemical shift artifacts. Jacoby *et al.* 27 implemented chemical shift imaging to provide signal contributions from all fluorine nuclei in PFOB to generate artifact-free ^19^F MR images. However, the *in vivo* detection sensitivity of 50.5% w/w PFOB NE was only 37% compared to 10% w/w PFCE NE. Yang *et al.* 38 selected PFCE as the objective was not only to deliver the Osimertinib drug but also to conduct real-time monitoring of PFCE NE bioaccumulation in the tumor tissue by the ^19^F MRI technique. PFCE has 20 magnetically equivalent fluorine atoms that result in a single peak on NMR spectra. This increases the signal-to-noise ratio and results in high-contrast ^19^F “hot spots” during clinical MRI, which can be placed within the anatomical context using ^1^H MRI. One concern raised by Flögel and colleagues regarding the biomedical use of PFCE was the occurrence of transient spleen enlargement after injecting a high *i.v* dose 27. However, in their study, a single *i.v* dose of PFCE NE resulted in a high PFC load (14 g of PFC/kg in mice). For drug delivery applications, a low *i.v* dose of PFCE NE is injected, which results in minimal PFC load in major organs 38, 54. For example, a single *i.v* injection of Osimertinib-loaded PFCE NE corresponded to ~2 g of PFCE/kg in mice. Further, no signs of tissue necrosis or inflammation were observed even after two weeks, as confirmed by hematoxylin and eosin staining of major organs. Nevertheless, long-term studies observing for signs of splenomegaly or monitoring the changes in aspartate transaminase and alanine transaminase levels (markers of liver damage) are required, especially when planning multi-dosing experiments with PFCE NEs.

Historically, PFC NEs were used to deliver oxygen to the lungs through liquid-assisted ventilation. This has inspired the use of PFC NEs for pulmonary drug delivery applications. Systemic drug delivery to the injured lung fails to achieve the desired drug concentration as the blood flow is redirected away from the site of injury. Pulmonary drug delivery overcomes this challenge and helps to achieve increased local concentrations of drugs without any global systemic exposure 18, 46. Ding *et al.* 18 selected PFOB, a low vapor pressure (10.4 torr at 37 °C) PFC, to formulate a NE for delivering siRNA targeting STAT3 through the intratracheal route. As per the literature, PFCs with low vapor pressure (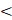 20 torr) do not increase pulmonary residual volume or compromise lung compliance 56. However, in addition to low vapor pressure, the spreading coefficient of the PFCs is also a critical parameter that needs to be taken into consideration for achieving wide intrapulmonary distribution of the therapeutic payload. For example, even though PFD has a low vapor pressure (13.6 torr at 37 °C) it is not a suitable candidate for pulmonary delivery as it has a low spreading coefficient of -1.5 dyne/cm 57. This would limit PFD from penetrating mucus-filled, collapsed, and under-ventilated areas of the lungs. Thus, the success of pulmonary drug delivery relies on identifying PFCs with a high spreading coefficient that can deliver payloads to lower lung lobes without triggering transient respiratory depression.

PFCs can be selected for specific applications based on their suitability. Ultrasound-triggered drug release is investigated as a non-invasive approach for achieving spatiotemporal control over drug delivery by using ultrasound waves as an external trigger. An ultrasonic wave is a longitudinal pressure wave with a frequency above the human audible range 58. PFC NEs are developed as “phase-shift microbubble precursors” to overcome limitations of PFC gas-filled microbubbles like poor drug loading and short circulation time (minutes) 15, 59. However, the developed PFC NE should be ultrasound-responsive, remain stable in the blood circulation, and release the therapeutic payload at sub-ablative ultrasound energy levels to prevent tissue damage 59. Rapoport *et al.* 40 used a low-boiling-point (29 °C) PFC such as Perfluoropentane **(PFP)**, as it requires low levels of ultrasound energy to trigger vaporization. Although PFP NE droplets might be expected to vaporize prematurely at physiological temperature (37 °C) before reaching the intended site, the authors theoretically showed an inverse relationship between the temperature required for PFP NE vaporization and the droplet diameter. As the droplet diameter decreases, the Laplace pressure on the PFC core increases, consequently increasing the boiling point of PFP. The group further supported their theoretical findings with *in vivo* experimental data, showing that PFP NE undergoes a droplet-to-bubble transition, also known as acoustic droplet vaporization (AVD), under ultrasound exposure. This facilitates an increase in surface area, causing the “ripping off” and release of Paclitaxel in ovarian carcinoma tumors without any unwanted bioeffects. Although the authors selected PFP based on its low boiling point, they did not comment on the vaporization efficiency of PFP (*i.e.,* how many microbubbles are formed from nanodroplets under the given ultrasound parameters and time), which has been identified as a critical parameter for low boiling point PFCs determining the amount of drug release 60.

PFCs are also selected to increase the stability of NEs, which is primarily compromised by a destabilization mechanism known as Ostwald ripening. Ostwald ripening is a molecular diffusion phenomenon in which the large droplets grow at the expense of the small droplets, leading to an irreversible increase in droplet diameter and ultimately phase separation. According to Lifshitz, Slyzov, and Wagner (LSW) theory, the thermodynamic driving force for Ostwald ripening is the increased water solubility of the dispersed phase as the droplet radius decreases. PFCs having high molecular weight and low continuous-phase solubility are reported to decrease Ostwald ripening rates in PFC NEs 61. Patel *et al.* 62 selected perfluorinated polymers like PFPE to formulate 7.24% w/v PFPE NE with celecoxib and monitored changes in the droplet diameter using dynamic light scattering (DLS) over a period of three months as an indicator of Ostwald ripening. Their data showed no changes in the droplet diameter at 4 °C (refrigeration temperature) and 25 °C (room temperature), supporting the use of PFPE to formulate PFC NEs with long-term colloidal stability. In another example, Ashrafi *et al.* 41 systematically screened twenty-seven PFCs to show a direct correlation between the observed evaporation rate of PFC and the colloidal stability of PFC NE when the Pluronic surfactant system remained constant. The authors selected PFTBA with an evaporation rate less than 0.01 g/h to formulate volatile anesthetics delivering PFC NE. PFTBA/isoflurane NE batches stored at room temperature over a period of 360 days showed 4% decrease in droplet diameter with no significant loss in isoflurane content. It is, however, important to emphasize that the stability of PFC NEs is not solely determined by the properties of PFCs. Other variables, such as surfactant properties, surfactant concentration, and the manufacturing process, also need to be optimized to achieve PFC NEs with long-term stability. Taken together, the selection of PFC core is application-specific, and a formulator has to evaluate several factors, including biological half-life 16, ability to develop artifact-free ^19^F MRI images 17, desired route of administration 46, intended application 19, 40, and potential to improve the stability of PFC NE 62, 63 during selection.

### 2.2 Selection of Surfactants

A surfactant is an amphiphilic molecule that reduces the interfacial tension to facilitate droplet break-up and subsequently stabilizes the newly formed interfaces to prevent recoalescence 14, 64. Older literature emphasized that the colloidal stability of PFC NEs is governed by the properties of PFCs rather than the emulsifier 61. However, recent literature contradicts this, as PFC NEs emulsified with surfactants varying in their hydrophilic-lipophilic balance **(HLB)** values at the same PFC-to-surfactant ratio and manufacturing conditions showed different colloidal stabilities over time 65, 66. For formulating PFC NEs as drug delivery carriers, researchers have selected surfactants that were initially used to develop FDA-approved PFC NEs for oxygen delivery. Consequently, the selected surfactants can be categorized into (1) non-ionic surfactants 51, 62, 67 and (2) lipid-based surfactants 46. This preference may be attributed to the available empirical data demonstrating their effectiveness to stabilize PFC in water, their proven biocompatibility in preclinical studies, and their hydrophilic nature, as indicated by their high HLB values (> 8). Surfactants with high HLB values favor the formation of PFC-in-water NEs rather than water-in-PFC NEs. However, a few recent studies have explored a new class of fluorinated polymeric surfactants 34, 68, synthesized to be compatible with the PFC core (for chemical structures, refer to **Figure 3**).

### (a) Non-ionic surfactants

The first generation of FDA-approved PFC NEs for oxygen delivery were stabilized using water-soluble triblock copolymers of poly(ethylene oxide) **(PEO)** and poly(propylene oxide) **(PPO)**. Pluronic F-68 (PEO_76_PPO_29_PEO_76_) was the first non-ionic emulsifier used in Fluosol^®^ 9. Pluronics consist of a hydrophobic PPO block that anchors at the droplet surface and two hydrophilic PEO blocks that extend into the aqueous phase for steric stabilization. These stabilizing PEO chains extend to form shells of nanometer thickness (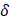). When two droplets approach at a separation distance (h) smaller than two times the thickness (h 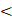 2δ), the chains experience strong repulsive forces to avoid loss in configurational entropy at the overlapping region 69. However, repeated administration of Fluosol^®^ triggered mild flu-like symptoms, attributed to the F-68 dose-dependent immune activation 70. Therefore, research efforts are directed towards investigating other biocompatible polymeric surfactants that can emulsify PFC in water. Sletten and her team published a polymer library of amphiphilic diblock and triblock poly(2-oxazoline) **(POx)** copolymers designed to mimic F-68 based on their structure-property relationships 65, 66. For triblock polymers, the hydrophilic PEO block in F-68 was replaced with either a poly(2-methyl-2-oxazoline) **[P(MeOx)]** block or a poly(2-ethyl-2-oxazoline) **[P(EtOx)]** block, and the hydrophobic PEO block was replaced with a poly(2-nonyl-2-oxazoline) block **[P(NonOx)]**. The hydrophilic and hydrophobic block lengths of POx polymer were controlled to be comparable to the ratio in F-68. The authors systematically showed that the droplet size stability of PFC NEs stabilized with triblock POx surfactants increases with an increase in hydrophilic block length, which favors steric stabilization. Note that in this study, the total PFC content (7:3 PFD/PFTPA) was 10 vol% and required 2.8 wt% of triblock POx emulsifier to formulate colloidally stable PFC NE. The total PFC content in Fluosol^®^ was 20 vol% (7:3 PFD/PFTPA) and was emulsified with 2.72 wt% of F-68 9. This means that, compared to F-68, a higher POx surfactant concentration might be required to emulsify the same amount of PFC in the formulation. Further, the authors compared the relative surface protein adsorption on PFC NEs emulsified with POx polymers to that with a polymer having poly(ethylene oxide) **(PEG)** as a hydrophilic block. Their Bradford assay data showed no significant differences in total protein adsorption, indicating POx polymers can effectively shield non-specific protein interactions to offer a stealth effect 66. Thus, POx polymers can be selected to formulate antifouling PFC NEs with prolonged circulation times. One avenue to explore is whether PFC NEs stabilized with POx polymers could be used as a safe alternative for delivering drugs to human populations positive for anti-PEG antibodies. Non-ionic polymeric surfactants can also be designed to leverage unique microenvironments within cells. The same research group showed the development of POx amphiphiles with disulfide linkers (P(MeOx)_27_-SS-P(NonOx)_8_). This reduction-sensitive surfactant was designed to respond to the increased concentrations of glutathione **(GSH)** within cells (2-10 mM) compared to the extracellular fluid (0.02-0.1 mM). The presence of high GSH led to the irreversible cleavage of disulfide bonds at the liquid-liquid interface. Consequently, demulsifying PFC NEs and releasing the therapeutic payload intracellularly 51. In another example, PFCE NE was formulated using poly(ethylene oxide)-co-poly(d,l-lactide) (PEG-PDLA), a biodegradable and pH-sensitive surfactant, to promote the endo-lysosomal escape of paclitaxel-loaded PFCE NE in tumor cells 67.

Another direction in the field is to use Pluronics^TM^ triblock copolymers with lower HLB values as compared to F-68 71. This selection criteria is particularly applied for stabilizing triphasic PFC NEs with hydrocarbon oil. The HLB scale provides a numerical value to characterize the balance between the hydrophilic and lipophilic groups in a surfactant and indicates the solubility of the surfactant in either the oil or water phase. On the other hand, hydrocarbon oils have required HLB values typically in the 10-15 range, which should be matched by the selected emulsifiers to develop colloidally stable NEs with water as an external phase 72. F-68 is primarily a hydrophilic surfactant with a high HLB value of 29 and is not an ideal candidate for emulsifying hydrocarbon oils 71. Therefore, pluronics with relatively low HLB values, such as pluronic P105 (HLB = 15), are selected to match the required HLB values of the selected oil. Additionally, as the HLB value of an individual surfactant may not always directly correspond with the required HLB value for oil, different non-ionic surfactants with varying HLB values are selected and blended in different ratios such that the HLB of the surfactant blend matches the targeted HLB requirement of oil 55. While the HLB value of non-ionic surfactants serves as a good metric to narrow down the selection list of emulsifiers, this semi-empirical approach is limited to triphasic PFC NEs. This is because, unlike hydrocarbon oils, PFC liquids do not have the required HLB values. Consequently, the selection of non-ionic surfactants for biphasic PFC NEs depends on conducting extensive screening experiments with different surfactants. Furthermore, PFC NEs stabilized with triblock copolymers are reported to maintain their colloidal stability in the presence of serum proteins 55, 73, 74. Recently, Zhalimov *et al.* 75 demonstrated that the amount of plasma protein adsorbed on the surface of the PFC NE droplets stabilized with triblock copolymers was directly proportional to the 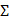
PEO/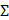
PPO ratio, rather than other parameters like molecular weight and length of the PEO and PPO block lengths.

### (b) Lipid-Based Surfactants

**Figure 4:**
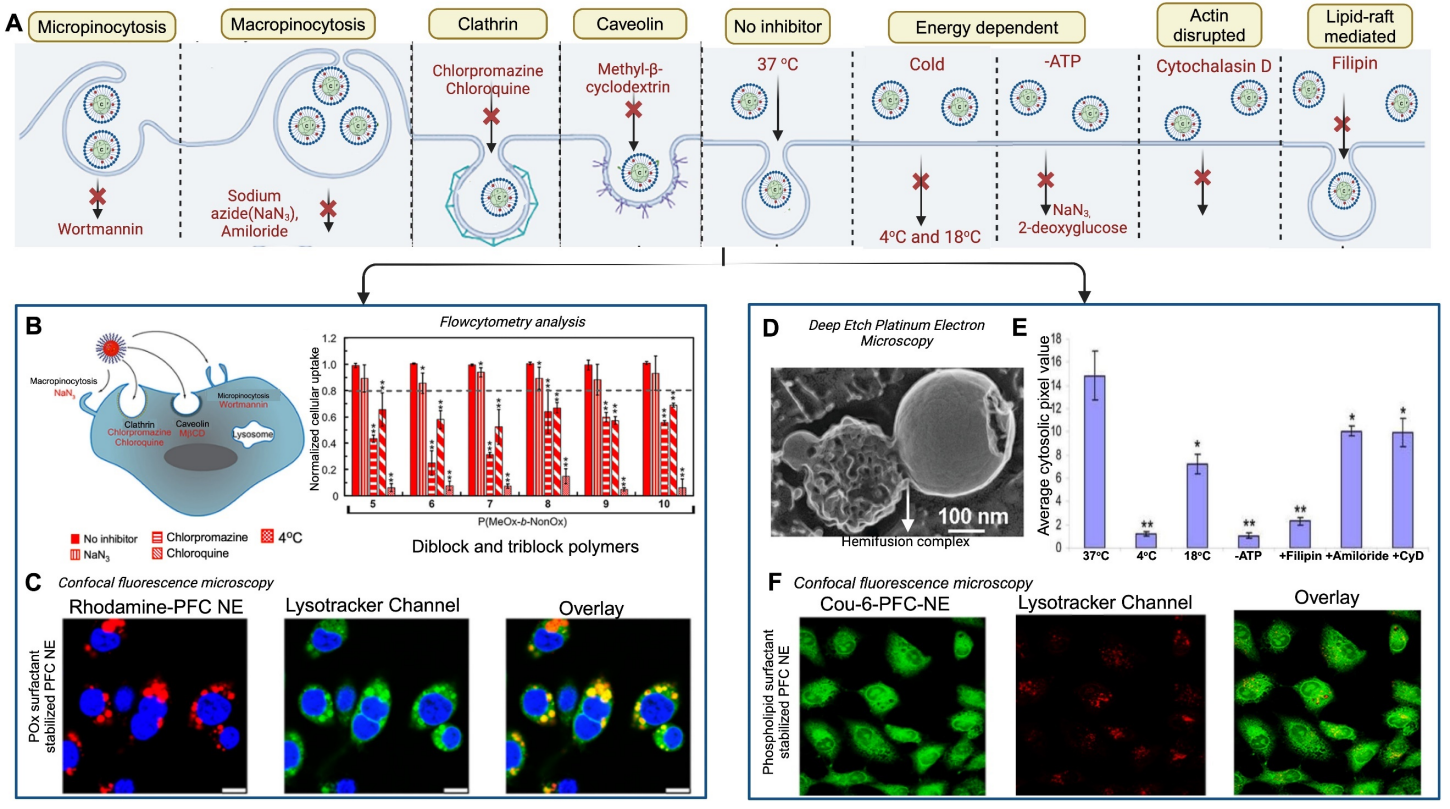
**Influence of surfactants on the uptake of PFC NEs. A.** Schematic representation of cellular uptake and commonly selected inhibitors for inhibiting each uptake mechanism. Created with Biorender.com. **B.** Quantified FACS analysis of the uptake of rhodamine-labeled PFC NEs emulsified with non-ionic surfactants. RAW264.7 macrophages were treated with the listed inhibitors. **C.** Confocal microscopy of RAW264.7 macrophages showing the uptake of PFC NEs emulsified with POx polymer. Rhodamine labeled PFC NE (red, Ex 532nm), LysoTracker (green, Ex 488nm), and nuclei stained with Hoescht (blue, Ex 405nm). Scale bar = 7.5 μm. Figures B and C adapted with permission from 66. Copyright (2020), American Chemical Society. **D.** Deep-etch platinum electron microscopy showing a hemifusion complex between the EYP stabilized PFC NE droplet and liposome (model bilayer membrane). Reprinted with permission from 78. Copyright (2008), American Chemical Society. **E.** Analysis of pixel intensities within the C32 melanoma cell cytosol in the presence or absence of inhibitors. Adapted from 80 with permission. Copyright (2008), Elsevier. **F.** Confocal microscopy in H9c2 cells shows the uptake of PFC NEs emulsified with phospholipids. Coumarin-6-labeled PFC NE (green) and LysoTracker dye (Red) visualized on a 60 × objective lens; scale bar = 10 μm. Adapted from 46 with permission. Copyright (2021), Elsevier.

The second generation of FDA-approved PFC NEs were sterically stabilized using lipid-based surfactants such as egg yolk phospholipids **(EYP)** (HLB = 10) and hydrogenated soybean phospholipid (HLB = 9) 42. Phospholipids are amphiphilic molecules composed of a phosphate group (polar head) and fatty acid chains (nonpolar tails) connected by a glycerol backbone 76. Based on the literature, phospholipids are preferred when the aim is to effectively deliver biologics intracellularly without exposing them to the acidic pH of endosomes 46, 49, 77. For PFC NEs, the selected surfactant dictates the cell uptake mechanism 66, 77. Mechanisms of cell uptake can be investigated using different inhibitors as shown in** Figure 4A.** Estabrook *et al.* 65 showed PFC NEs stabilized with POx or Pluronic F-68 surfactants are primarily internalized through endocytosis, regardless of droplet diameter (150-300 nm), in both phagocytic (RAW 264.7 macrophages) and non-phagocytic (A375) cell lines. Day *et al.* 66 corroborated these findings by showing colocalization of the rhodamine-labeled PFC NE droplets with Lysotracker green dye, which selectively labels acidic cell organelles like endosomes and lysosomes. Further, clathrin-dependent endocytosis was the dominant route of internalization for PFC NEs emulsified with non-ionic surfactants, as a 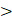 40% decrease was observed in the presence of chlorpromazine, an inhibitor of clathrin-mediated endocytosis **(Figure 4B and C)**. In contrast, Soman *et al.* 78 showed that PFC NEs emulsified with EYP (droplet diameter: ~240 nm) avoid endosomal uptake and deliver therapeutic cargo directly to the cytoplasm through 'contact-facilitated drug delivery'. The authors performed deep-etch platinum replica electron microscopy to investigate a mechanism that depends on lipid mixing between the surfactant monolayer and the bilayer membrane to form a hemifusion complex **(Figure 4D)** 49. Importantly, molecular dynamics simulations supported the role of the PFC core in the reorientation and protrusion of the hydrophobic tails of the surfactant 79. The formation of the hemifusion complex enabled direct shuttling of the cargo to the cell membrane, which was further trafficked intracellularly via an energy-dependent process known as caveolae/lipid raft-mediated internalization 49, 78, 80. Lipid rafts are dynamin-dependent, cholesterol-sensitive membrane domains that are intracellularly trafficked to caveolin-1-positive endocytic compartments, which are at a neutral pH, unlike acidic early endosomes 81. The research group used a panel of cell uptake inhibitors and showed that cell uptake was five-fold lower in the presence of filipin, which specifically inhibits lipid raft formation by binding to membrane cholesterol **(Figure 4E)** 80. Recently, Qin *et al.* 46 showed minimal overlap of the fluorescence signal between the fluorescently labeled Cou-6-PFOB NE emulsified with phospholipid (~150 nm) and the LysoTracker Red DND-99 dye **(Figure 4F)**. Furthermore, the group developed protein-phospholipid complexes **(PPCs)** between HSPC and pigment epithelium-derived factor (PEDF), a glycoprotein that protects against cellular oxidative stress. PPCs increase the lipophilicity of the therapeutic proteins, thereby increasing protein loading in the PFOB NE scaffold.

One factor to consider during formulation is that lipid-based surfactants are susceptible to oxidative degradation due to the presence of unsaturated fatty acid chains within phospholipids. This compromises their functionality to stabilize interfaces 82, which negatively impacts the storage stability of PFC NEs. To address this, Qin *et al.* 46 incorporated a low concentration of 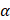-tocopherol in the PFC NE formulation to prevent oxidative degradation of phospholipids. Tocopherol is an effective interfacial antioxidant that prevents the oxidation of phospholipids due to its free radical scavenging activity at interfaces 83. However, the group did not present data demonstrating the long-term stability of the formulated PFC NE or the effectiveness of tocopherol at PFC/water, which requires further investigations.

### (c) Semifluorinated Polymeric Surfactants

Semifluorinated polymeric surfactants represent a new class of amphiphilic polymers composed of hydrophilic head groups and fluoroalkyl tails. Initially, these surfactants were used to formulate PFC NEs for *i.v* delivery of fluorinated anesthetics to induce rapid anesthesia compared to inhalation. However, the presence of large fluorophilic block triggered allergic responses due to histamine release in large animal studies, resulting in failures of these PFC NEs 84. However, recent advances in synthetic polymer chemistry have supported the development of fluorinated polymers with different chemical structures for biomedical applications 85. Decato *et al.* 86 synthesized a series of semifluorinated polymers and showed that the total fluorine content of the surfactant determines the initial droplet diameter and long-term stability of PFOB NEs. Polymers composed of three perfluoro-tert-butyl (PFtB_TRI_) groups as the fluorophilic tail resulted in PFOB NE with a smaller droplet diameter and a decreased Ostwald ripening rate as opposed to the polymers with a single PFtB group (PFtB_MONO_), irrespective of the chain length of the hydrophilic monomethyl poly(ethylene glycol) (mPEG) head group. This difference in PFOB NE stability was explained by determining the critical micelle concentration **(CMC)** based on surface tension measurements. The mPEG_x_-(PFtB_MONO_) polymers did not drive the self-assembly to form micelles. In contrast, mPEG_x_-(PFtB_TRI_) polymers showed acceptable CMC values below 1 mM, driving self-assembly due to the increased interaction between the fluorous tails on the polymer. Nevertheless, the scope of using semifluorinated amphiphiles is limited, as volatile fluorinated anesthetics that solubilize in PFC cores can be delivered through these systems. To broaden their applicability for loading hydrophobic drugs, further work should explore whether an intermediate hydrocarbon segment can be incorporated into the surfactant design and the implications of this strategic installation on surfactant properties such as CMC values.

Another notable example in this category is fluorinated polyethyleneimine **(F-PEI)** surfactants, specially synthesized to deliver siRNA for achieving sequence-specific gene silencing. Polycationic polymers like PEI condense siRNA through electrostatic interactions to form polyplexes. Polyplexes provide superior transfection efficiency by facilitating the endosomal escape of siRNA through the proton-sponge mechanism, which is an osmosis-driven process triggered by the proton buffering capacity of PEI. However, the use of PEI is limited due to its cytotoxicity 87. Chen *et al.* 68 proposed that formulating PFD NE stabilized with F-PEI would minimize the cytotoxicity of PEI. To test their hypothesis, the research group first synthesized F-PEI by grafting the fluoroalkyl chains from heptafluorobutyric anhydride onto the primary and secondary amines present in PEI. In the second step, F-PEI was used to emulsify PFD (FPEI@ PFD NE). Following this, the process of siRNA condensation on F-PEI@PFD NE was driven by low-speed vortexing. The cell viability data supported their hypothesis, as F-PEI@PFD NE was significantly less toxic compared to naïve PEI even at high w_PEI_/w_siRNA_ ratios owing to the decreased PEI mobility 87. Another important aspect of their work was that the F-PEI@PFD NE (~150 nm) had the same buffering capacity as naïve PEI and condensed siRNA at the same w_PEI_/w_siRNA_ ratio, indicating that covalent modification of amine groups on PEI with fluoroalkyl tails did not compromise polycation functionality. However, the colloidal stability of the formulated F-PEI@PFD NE stored at 4 °C was monitored for 7 days. In a comparable study, Lv, Jia *et al.* 88 used the same synthetic chemistry to develop F-PEI as Chen *et al.* and showed that under aqueous conditions, the amide bond linking the fluoroalkyl tails to PEI undergoes hydrolysis within 10 days. Therefore, the study duration for monitoring the stability of F-PEI@PFD NE should be extended beyond 10 days to observe if the stability of F-PEI@PFD NE is compromised due to F-PEI hydrolysis or whether the presence of a hydrophobic PFD core protects the embedded amide bond from hydrolytic degradation. In another example, Gao *et al.* 89 synthesized a fluorinated cationic polymer (C_11_F_17_-PBLA-DET) by performing an aminolysis reaction of C_11_F_17_-poly(β-benzyl-l-aspartate) (C_11_F_17_-PBLA) with diethylenetriamine (DET). PFP was emulsified with C_11_F_17_-PBLA-DET, followed by coating with PGA-g-mPEG to form nanodroplets capable of delivering nucleic acids to the cancer cells. Although semi-fluorinated surfactants open new avenues in drug delivery science, several critical aspects need further investigation, including surfactant characterization, the ability to scale up synthesis, and proving preclinical efficacy and safety.

After surfactant selection, the next step is to determine the optimal surfactant concentration required for formulating PFC NEs. This relies on optimization experiments, regardless of the surfactant type 34, 66. The required surfactant concentration is selected based on achieving the predefined goals, such as targeted droplet size, low dispersity, long-term droplet size stability of PFC NEs under the set processing conditions.

### 2.3 Selection of Hydrocarbon oil: A Key Component in Triphasic PFC NEs

The developers of Oxyfluor^TM^ (HemaGen/PFC Ltd., MO, USA) first reported increased storage stability of PFC NEs formulated with 2% w/v soybean oil compared to PFC NE formulated without oil. The increased stability was attributed to the long-chain triglyceride, which covers the dispersed fluorous phase to improve the adsorption of hydrophobic surfactant tails 70. In 1992, the company patented this three-liquid-phase system innovation 90. Later studies have reported similar findings, as increased stability of PFC NEs was observed in the presence of hydrocarbon oil 71, 91. However, recent efforts are directed towards repurposing this small percentage oil (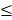
5% w/v) phase to incorporate hydrophobic small molecules (~1000 Da) and lipophilic dyes (*e.g*., carbocyanine dyes) as a second imaging modality 17, 34, 55. Consequently, the selection of hydrocarbon oil primarily depends on the solubility of the hydrophobic drug in the oil phase, as it directly affects the amount of drug loaded in triphasic PFC NEs. For instance, Herneisey *et al.* 39 performed solubility studies to determine the solubility of Resveratrol, a natural antioxidant, in Miglyol 812, propylene glycol, and olive oil. The authors selected propylene glycol based on the maximum solubility of resveratrol, an anti-oxidant drug, compared to the other two tested oils. Additionally, the presence of the oil phase allows the formulators to incorporate FDA-approved solubilizers (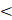
1% w/v) if required to increase the solubility of the hydrophobic drugs. For example, in another study, diethylene glycol-monoethyl ether (Transcutol^®^ HP) was incorporated in a small percentage along with Miglyol 812 to increase the amount of celecoxib, a non-steroidal anti-inflammatory drug, that can be loaded into the PFC NE 74.

In addition to the rational selection of formulation components and determining the required concentration of each component, an emulsion scientist must carefully select the manufacturing technique and optimize processing parameters, as they influence the physicochemical properties of PFC NEs 74. Therefore, the following sections will focus on the manufacturing aspect of PFC NEs.

## 3. Manufacturing of PFC NEs for Drug Delivery Applications

**Figure 5:**
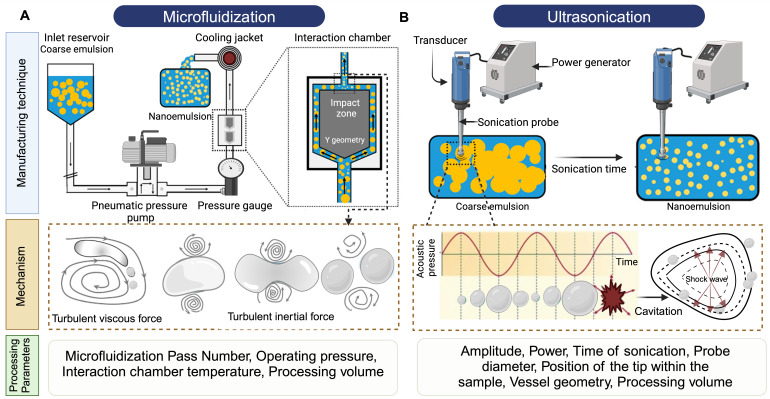
**Manufacturing techniques for developing PFC NEs**. Illustrative figure showing instrumentation, mechanism of droplet breakup and processing parameters for **A.** Microfluidization (MF) and **B.** Ultrasonication emulsification (UE). Created with Biorender.com.

The process of manufacturing PFC NEs can be divided into two steps. The first step is the formation of a coarse emulsion, typically achieved by low-speed vortexing or magnetic stirring of the components. The second step is focused on droplet size reduction, either by microfluidization **(MF)** or ultrasonication emulsification **(UE).** For instrumentation, mechanism of droplet break-up, and processing parameters refer to **Figure 5**. Although both the homogenization techniques are categorized under high-energy methods (input energy density: ~10^8^-10^10^ W kg^-1^) 92, the mechanism of droplet breakup differs between the two techniques 93. In general, NEs can also be manufactured using low-energy techniques (input energy density: ~10^3^ W kg^-1^). However, the popularity of selecting high-energy methods is because of the high interfacial tension at the PFC/water interface (50-60 mNm^-1^) that opposes the dispersion of PFC in the aqueous phase as compared to the HC oil/water interface (30-40 mNm^-1^) 4. Therefore, in this section, we will focus on MF and UE, two commonly reported techniques for manufacturing PFC NEs.

### 3.1 Microfluidization

In microfluidizers, the coarse emulsion enters the system through an inlet reservoir and is accelerated by using a high-pressure pneumatic pump capable of generating operating pressure as high as 40,000 psi. The coarse emulsion is then directed through a microchannel with an axially varying geometry, either Y-type or Z-type, often referred to as an interaction chamber. The enclosure of this interaction chamber is made of stainless steel, while the interior is either aluminum oxide ceramic or polycrystalline diamond, which offers chamber wear resistance. For liquid-liquid dispersions, such as NEs, a Y-type interaction chamber with microchannel diameters ranging from 75
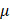
m to 125
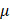
m is generally preferred to achieve a narrow-size distribution. Furthermore, due to the high level of turbulence, an auxiliary processing module (APM; also known as a return pressure chamber) can be used in conjunction with the interaction chamber. For a Y-type interaction chamber, a Z-type AMP is installed downstream to add backpressure. This prevents interaction chamber wear-off and stabilizes flow rate 94, 95. In the interaction chamber, the process of droplet breakup occurs due to turbulent viscous **(TV)** and turbulent inertial **(TI)** forces. As the fluid is accelerated, the droplets experience turbulent flow fields and interact with short-lived eddies. The nature of the interaction between the droplet and the eddies depends on the droplet diameter (d) and the length scale of the eddies (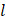). If the droplet diameter is smaller than the length scale of the eddy (d 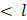), the droplet experiences viscous shear at the interface because of the velocity gradient created by the eddy. Depending on the strength of the viscous gradient and the time scale of the droplet-eddy interaction, the droplet undergoes elongation and eventually breaks up. This process is referred to as the TV droplet breakup. In contrast, for droplet breakup by TI forces, short-length scale eddies (d 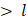) with low kinetic energies participate in generating pressure fluctuations at the interface 96. Following a fixed number of microfluidization passes through the interaction chamber, the sample exits through an outlet, where heat exchangers or cooling coils can be installed to prevent overheating of PFC NEs. The microfluidization process established on a lab scale can be linearly scaled to pilot or commercial scale by using interaction chambers consisting of parallel arrangements of multiple fixed-geometry microchannels. This design approach helps to achieve shear and pressure profiles similar to those obtained during lab-scale manufacturing 97. A few studies have shown implementation of MF technology to scale up (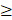 50 mL) drug-loaded PFC NEs 41, 55, 98.

In microfluidizers, the operating pump pressure and number of passes are the critical processing parameters **(CPPs)** primarily governing the measurable critical quality attributes **(CQAs)** of PFC NEs, such as droplet diameter, dispersity index, and long-term droplet size stability 74. However, there is an interplay between the processing parameters and the composition of PFC NE. For example, Herneisey *et al.* 74 reported that changing formulation compositions of triphasic PFC NEs resulted in varying CQAs despite being processed under the same homogenizing conditions. Although a systematic study investigating the influence of different operating pump pressures on the CQAs of PFC NEs has not yet been reported in the field, the reported operating pressure for manufacturing biphasic or triphasic PFC NEs on the lab scale and pilot scale models (M110S, M110P, and M110EH) lies between 10,000 psi and 20,000 psi. Liu *et al.* 98 formulated PFC NEs with a fixed composition and demonstrated that the droplet diameter of PFC NEs decreases with an increase in the number of microfluidization passes at a constant operating pressure. It is, however, important to note that no significant decrease in droplet diameter was observed after increasing the number of passes beyond a critical number. This implies that increasing the number of passes to decrease the droplet diameter of PFC NEs applies until a size threshold is achieved. After that, the rate of surfactant adsorption can become a limiting factor, increasing the risk of overprocessing PFC NEs. The phenomenon of overprocessing occurs when the time scale of collision between the droplets is smaller than the time scale for surfactant adsorption, leading to an irreversible increase in droplet diameter 99. In general, another concern associated with high-energy homogenization techniques is the increased product temperature, which can lead to drug degradation. Mao *et al.* 100 in their work on M110EH, recorded an increase in NE temperature as the operating pressure was increased from 40 MPa to 120 MPa. However, their data showed that even at a high operating pressure of 120 MPa (~17,500 psi), the final product temperature was ~32 °C, owing to the high emulsification efficiency of microfluidizers compared to other high-energy methods. Although the NEs formulated by Mao *et al.* do not contain PFCs, studies conducted by other researchers on triphasic PFC NEs corroborated the findings 62, 98. Liu *et al.* recorded ~25 °C as the final product temperature when processed at an operating pressure of 15,000 psi for 6 passes on M110EH 98. This observation was primarily attributed to the design of microfluidizers, which allows the interaction chamber to be ice-cooled prior to manufacturing.

### 3.2 Ultrasonication Emulsification

The droplet breakup in UE is achieved due to sound waves with frequencies above 20 kHz. An ultrasonicator consists of a metal probe, an electric generator, and a piezoelectric transducer, which converts the electrical voltages into mechanical vibrations of the same frequency. The mechanical vibrations are amplified and directed through the metal probe to the tip submerged in the sample. This generates sinusoidal pressure fluctuations in the liquid and forms cavitation bubbles near the tip. The bubbles undergo a series of compressions and expansions before experiencing a catastrophic collapse to generate high shear forces that cause droplet breakup. This mechanism is also referred to as cavitation-induced emulsification. The generated acoustic pressure depends on CPPs like maximum amplitude, time of sonication, and frequency 101.

Bérard *et al*. 34 varied output amplitude settings from 20% to 80% on a 750 W power commercial ultrasonicator. Their results indicated that as the percent amplitude increases, the droplet diameter decreases. This can be explained as increasing amplitude proportionally increases the power emitted into the PFC NEs. However, this observation holds true only at lower surfactant concentrations, as at high surfactant concentrations the authors reported no changes in the droplet diameter, irrespective of percent amplitude. The result suggested that at high surfactant concentrations, the droplet diameter becomes insensitive to ultrasonication amplitude, similar to findings reported by other researchers in the NE literature 92, 102. The time of ultrasonication can be controlled by performing UE either in continuous mode or pulse mode. Throughout the PFC NE literature, researchers have preferred using pulse mode, perhaps to avoid overheating the final product. For example, Li *et al.* 103 performed ultrasonication with a 1.5 s power-on and 2 s power-off cycle for 30 min at a 30% output amplitude setting in an iced water bath to avoid overheating of PFOB NE. Although ultrasonication generally is performed by placing the vessel in a cold water bath 15, 45, researchers have used a sequential manufacturing approach to avoid subjecting thermosensitive payloads, especially biologics, to high temperatures during the initial stages 18. For example, Chen *et al.* 68 first emulsified PFD NE with a fluorinated polycationic polymer (F-PEI) through UE at 45% amplitude in pulse mode for 30 min on an ice bath. In the later step, the pre-formulated PFD NE with F-PEI as a chemical handle was vortexed at a low speed with siRNA to develop functional polyplexes demonstrating gene silencing activity.

Further investigations are required to determine how apparatus-specific parameters like the geometry of the vessel containing the coarse emulsion, probe diameter, position of the tip within the sample, and fluctuations in temperature of the water bath have any impact on the CQAs of PFC NEs. Although slight variations in these parameters may not significantly contribute to CQA changes during small processing volumes, they will significantly alter the CQAs during processing large-scale volumes (liters). Despite the limited knowledge in the PFC NE field on UE, a cited reason for selecting UE is its suitability for low processing volumes (1 to 5 mL) as opposed to MF (~25 mL). This is particularly beneficial during optimization experiments where limited monetary resources constrain the research project.

## 4. Potential Challenges in the Reported Literature on PFC NE

The first challenge is that information on the processing parameters is often inadequately reported in research articles. This compromises the reproducibility and creates barriers for integrating scientific findings in the field. For example, researchers report on the amplitude or the time of ultrasonication 68, but the details on the power of the ultrasonicator, diameter of the probe, vessel geometry, or name of the specific ultrasonicator model are often omitted. For MF, a few studies 16, 38 report the total time required for MF, while the other studies report the total number of MF passes or stokes required to achieve a targeted droplet diameter on a particular microfluidizer model 17, 55. Reporting on the total time for MF can provide comprehensive information, provided supporting details, such as the total volume processed during manufacturing or the time required per MF pass at a set operating pressure, are specified by the researchers.

**Figure 6:**
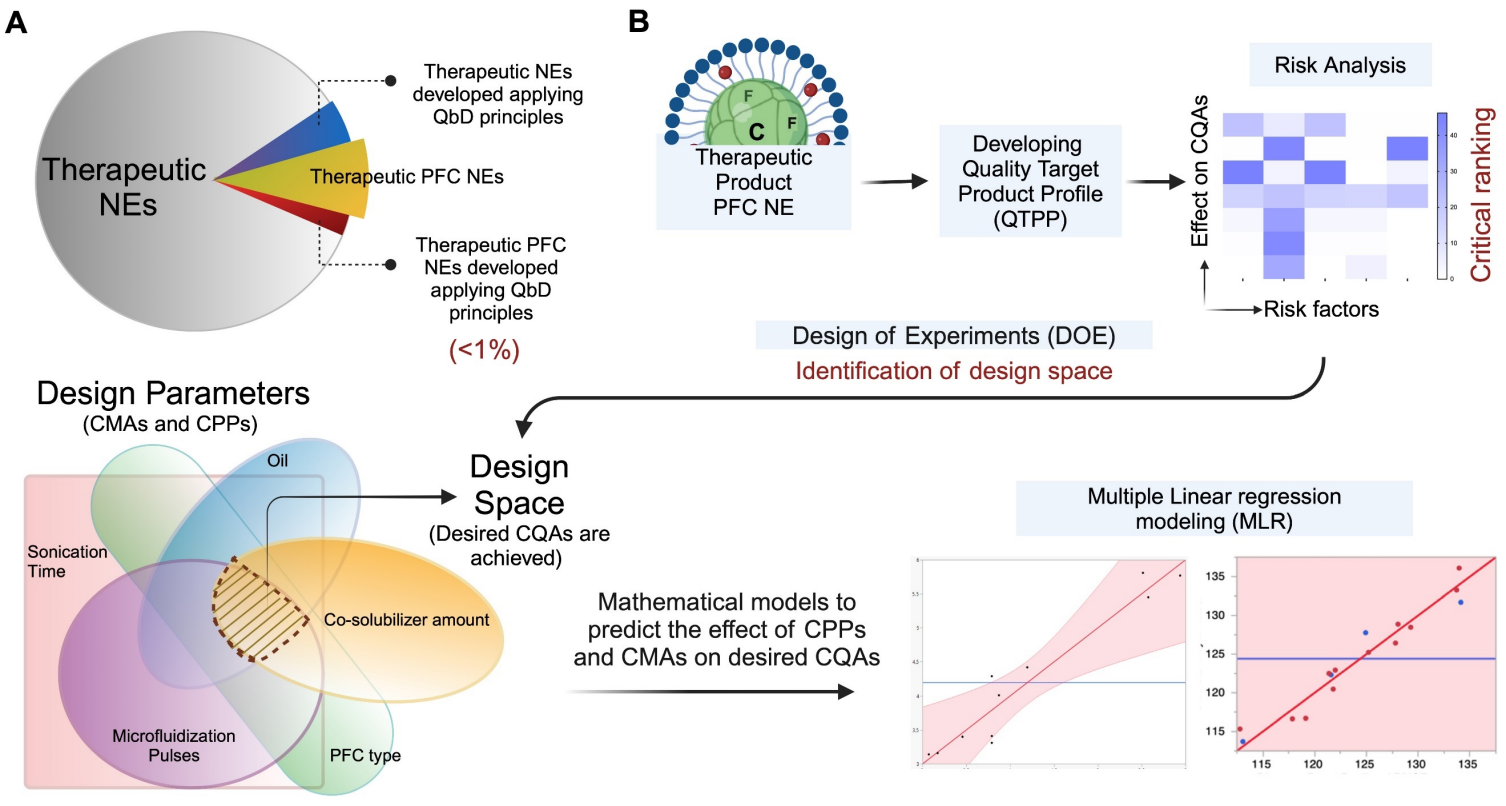
**Application of QbD for developing PFC NEs. A.** Academic landscape showing proportion of publication counts on therapeutic NEs and PFC NEs administered parenterally. Less than 1% of publication employed QbD approach for manufacturing therapeutic NEs or PFC NEs. Literature counts were obtained from Scopus^®^ using Keywords: Nanoemulsions; Perflurocarbon AND Nanoemulsions; Drug delivery; QbD. NEs delivered topically, intraocular, or orally were omitted. PFC NEs include both biphasic and triphasic NEs.** B.** Simplistic representation of QbD in PFC NE manufacturing. Created with Biorender.com.

The second challenge is that in current publications, emphasis is placed on conducting empirical assessments to validate the therapeutic efficacy of PFC NEs, while limited focus is given to understanding the manufacturing aspect of PFC NEs. However, during early development, it is recommended to simultaneously address questions such as: Does the selected manufacturing technique support the scale-up of the product? Which material attributes and processing parameters would influence the CQAs if the product was scaled up in the future? Recently, product development approaches like Quality by Design **(QbD)** have gained popularity for developing various nanoparticles. Nevertheless, the application of QbD for optimizing PFC NE composition and manufacturing parameters is limited **(Figure 6A)**. According to the ICH Q8 guidelines, the US FDA defines QbD as “a systematic approach to development that begins with predefined objectives and emphasizes product and process understanding and process control based on sound science and quality risk management” 104. For example, after demonstrating the therapeutic effectiveness of celecoxib-loaded theranostic PFC NE in an inflammatory pain rodent model 17, 73, Herneisey *et. al*. 74, 105 implemented the Quality by Design **(QbD)** approach as a next step to gain a thorough understanding of the manufacturing process and to define a design space for formulating PFC NEs with improved quality. The authors performed the first-ever risk assessment on triphasic PFC NEs to narrow down the high-risk material and processing parameters that would significantly impact the desired CQAs. This guided the authors to identify factors such as the number of microfluidization passes, type of PFC, and concentration of oil as a few of the critical processing parameters (CPPs) and critical material attributes (CMAs) that needed to be systematically investigated by performing design of experiments (DOE). DOE serves as a statistical tool that helps to systematically study and optimize the values of CMAs and CPPs such that specified CQAs are achieved within the design space. The authors concluded these studies by developing predictive multivariate linear regression **(MLR)** models to establish a relationship between the changes in the CPPs and CMAs and their effect on CQAs, such as droplet diameter, drug loading, and long-term stability **(Figure 6B)**. Overall, implementation of QbD principles helps to increase process understanding, guides researchers to develop PFC NEs with improved colloidal stability and drug loading and establishes control over the manufacturing process 74, 105. For an in-depth understanding of the basic elements of QbD and the need for QbD application in academic nanomedicine research, we direct the readers to read an excellent review 106. Currently, there are no approved PFC NEs for drug delivery on the market, but we speculate that following FDA-recommended pharmaceutical manufacturing approaches at the academic level to develop products that consistently meet target specifications can take the field one step closer to clinical translation. Additionally, in the future, this can prevent situations where manufacturing flaws of unidentified nature led to supply stoppages and shortages of nanomedicines like Doxil™ in the USA.

The third challenge is the need for performing robust characterization of PFC NEs developed for drug delivery applications. In majority of the publications, droplet diameter measured using DLS. Grapentine *et al.* 31 recommended using cryo-TEM as an orthogonal sizing technique for monitoring the long-term size stability of heat-sterilized PFC NEs stabilized with phospholipids. In contrast to DLS, which only provides averaged hydrodynamic diameter, cryo-TEM distinguished between PFC NE droplets and liposomes (PFC-free nanovesicles) formed during long-term storage. Thus, using techniques like cryo-TEM along with DLS can provide in-depth information on the droplet diameter changes without any bias. Recently, Janjic *et al.* 107 published a list of routine *in vitro* quality assessments for characterizing PFC NEs, which can be performed before *in vivo* testing. These tests include assessing droplet size stability after sterile filtration (0.22 μm filters), high-speed centrifugation (mechanical stress), thermal cycling, and post-exposure to high serum-containing biological media (biological stress). Few reports have also published accelerated stability testing conditions, where PFC NEs were subjected to extreme conditions such as elevated temperatures 41, 105. As compared to assessing longitudinal stability, the development of accelerated stability testing protocols for PFC NEs can save time and resources for manufacturers. Herneisey *et al.* in parallel developed an accelerated stability testing model and a shelf-life stability testing model to evaluate whether the accelerated stability model (7 days) can accurately predict changes in size, polydispersity index, and fluorescence signal loss of theranostic PFC NEs upon long-term storage (7 months). The authors showed that the accelerated stability testing model accurately predicted changes in CQAs in a time-efficient manner, speeding the identification of stable PFC NEs 105.

## 5. What's next? Targeted Perfluorocarbon Nanoemulsions for Drug Delivery

The next leap in the field is to design PFC NEs with targeting ligands for achieving precise spatial control over drug delivery. Currently, no therapeutic nanomedicines with an active targeting ligand are commercially available 108. However, relying on passive targeting mechanisms, such as the EPR effect or RES-mediated uptake, produces variable results between preclinical models and clinical trials 109. From a clinical perspective, the impetus for developing targeted nanoparticles as an innovative therapeutic intervention lies in their potential to alter biodistribution and pharmacokinetic profiles, reduce unwanted systemic exposure, and improve therapeutic efficacy 109. A variety of ligands, such as small molecules, antibodies, peptides, and aptamers, have been conjugated to the surface of PFC NEs to support active targeting.

**Figure 7:**
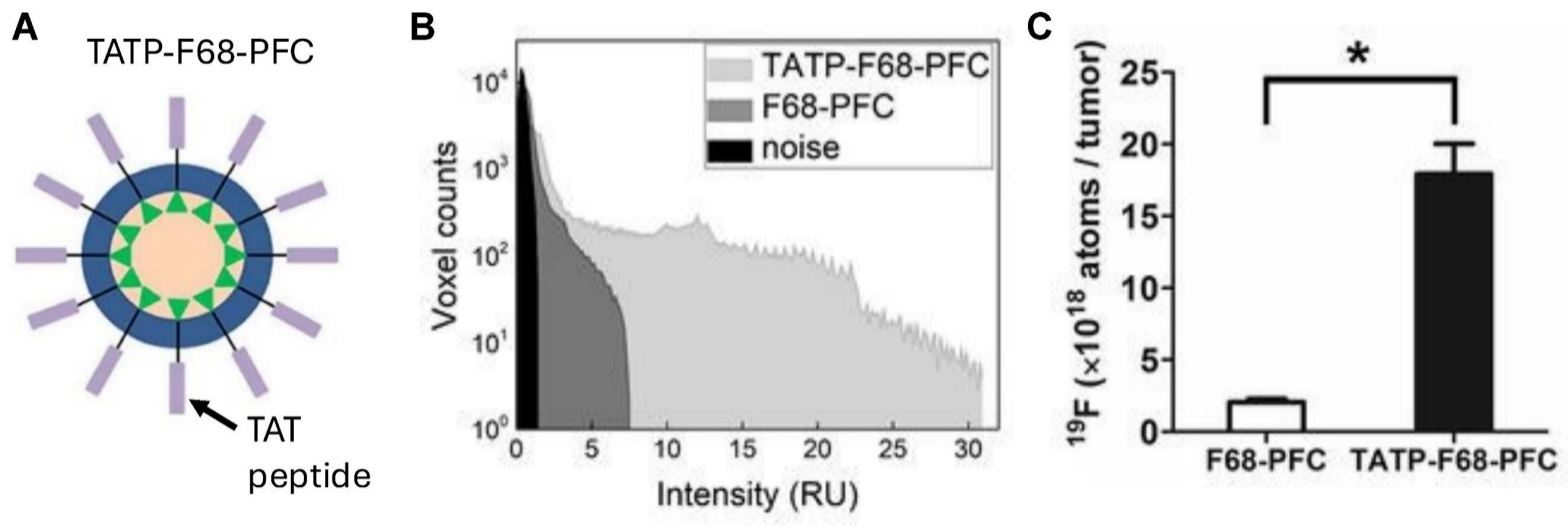
**A.** Schematic representation of TAT peptide surface-conjugated to PFCE NE droplet (TATP-F68-PFC) for increasing uptake in CAR T cells. *In vivo* ^19^F MRI signal enhancement in TATP-F68-PFC labeled human CAR T cells. **B.** A histogram of the ^19^F signal-to-noise ratio for each image voxel in the tumors is displayed and shows sensitivity improvement of the TATP-containing PFC NE compared to control. **C.** Comparison of apparent ^19^F atoms per tumor, measured *in vivo* for mice (n=4). * indicates p<0.001 for TAT-F68-PFC nanoemulsions compared to control. Figures adapted with permission from 111. Copyright (2020) John Wiley and Sons, Inc.

The targeting ligand enables ligand-mediated receptor interactions, which facilitates the binding of PFC NE droplets to the cell surface and intracellular transport via endocytosis. Several studies have demonstrated that increased intracellular drug delivery translates into improved biological outcomes compared to non-targeted PFC NEs 43, 55. For example, Vichare *et al.* 55 showed that the uptake of folic-acid-conjugated celecoxib-loaded PFC NE was significantly higher in LPS-activated macrophages (M_1_-like phenotype) overexpressing folate receptors (FR) compared to non-targeted celecoxib-loaded PFC NE. This increased uptake correlated with improved therapeutic efficacy, as increased suppression of proinflammatory cytokines (TNF-α and IL-6) was observed in these macrophages. Bae *et al*. 110 developed folate-PFC/rhodamine NEs to specifically target FR-positive tumors. The authors showed that folate-targeted PFC NE specifically accumulated in the FR-positive xenograft tumors in mice post-intravenous administration. In contrast, minimal accumulation was observed in the tumor tissue with non-targeted PFC NE. While this work did not incorporate a therapeutic agent, it shows that targeting can significantly improve the biodistribution of the drugs to the desired site with minimal off-target effects. In another example, Hingorani *et al.* 111 developed PFC NE displaying cell penetrating peptides (CPPs) derived from the transactivator of transcription **(TAT)** component of the human immunodeficiency virus type-1 to facilitate *ex vivo* labeling of engineered chimeric antigen receptor lymphocytes **(CAR T)** cells **(Figure 7A)**. CARs are synthetic receptors that redirect lymphocytes (T cells) to recognize and eliminate cells expressing a specific target protein or antigen. One of the limitations of CAR T cell therapy is its inability to track CAR T cell trafficking 112. *Ex vivo* labeling of CAR T cells can (1) allow for real-time monitoring of CAR T biodistribution, (2) help quantify the number of survival therapeutic cells, (3) guide optimization of the required dose, and (4) provide insights into off-target toxicities. However, two major technical challenges during *ex vivo* labeling of CAR T cells are their small cytoplasmic volume and their poor ability to perform phagocytosis, which limits the uptake of imaging probes by the CAR T cells. The group showed that engineering PFC NE droplets to display CPPs can overcome this challenge, as an 8.2-fold higher uptake of the imaging probe was observed in the therapeutic CAR T cells with TAT surface-conjugated PFC NE droplets compared to the unmodified PFCE NE droplets **(Figure 7B and C)**. Moreover, PFCE labeling of therapeutic CAR-T cells did not affect the phenotype or their functionality.

**Figure 8:**
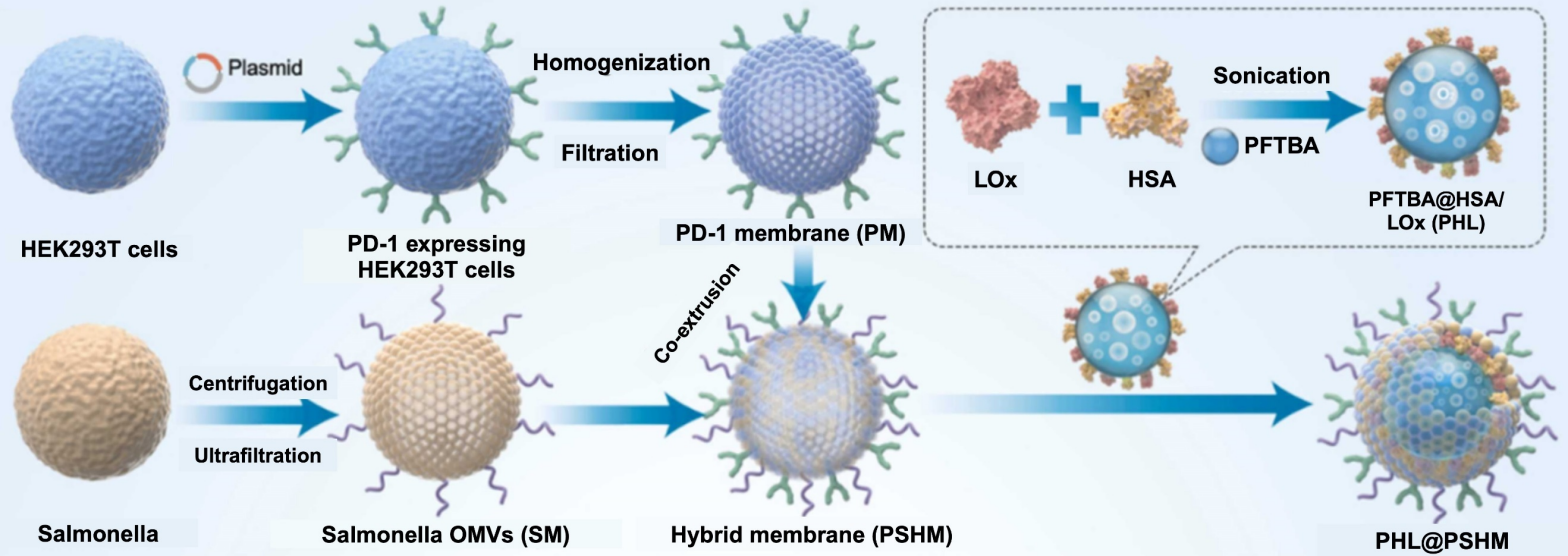
The preparation process of PHL@PSHM. HEK 293T cell line expressing a fusion protein PD-1/DsRed were amplified and homogenized to obtain the PD-1-expressed cell membranes (PM). Bacterial outer membrane vesicles (OMVs) were obtained from strain (VNP20009) of Salmonella typhimurium (SM). Hybrid membrane vesicles (PSHM NVs) were formed by co-extrusion. PFTBA@HSA/Lox nanoemulsion (PHL) was prepared by sonication of PFTBA and the aqueous solution of HSA and LOX. The final construct, PHL@PSHM was prepared by co-extruding PHL with PSHM. Reprinted with permission from 50. Copyright (2023) Elsevier.

Recently, hybrid cell membrane-coated PFC NEs have been developed as a successful strategy for site-specific accumulation of payloads 50, 113. For instance, Zhang *et al.* 50 formulated PFTBA NE loaded with lactate oxidase (LOX), which catalyzes the conversion of lactic acid to pyruvate and generates toxic hydrogen peroxide (H_2_O_2_) in cancer cells. The oxygen required for this reaction was provided by the PFTBA, as PFCs have excellent oxygen-carrying capacity. Next, the authors camouflaged PFTBA nanoemulsion using hybrid membrane coatings (PSHM) composed of outer membrane vesicles of Salmonella typhimurium (SM) and the membrane of PD-1-expressing HEK293T cells (PM) **(Figure 8)**. The group showed a three-pronged, synergistic therapeutic approach to inhibit tumor growth. First, by upregulating the expression of PD-L1 on the surface of tumor cells due to SM, thereby improving response to immune checkpoint inhibition. Second, by the targeted blocking of PD-L1 of tumor cells due to PM. Third, by boosting specific anti-tumor responses using photothermal therapy and generation of H_2_O_2_. The tumor growth inhibition rate of biomimetic PHL@PSHM plus laser radiation was as high as 92.8% compared to the PBS treatment in 4T1 breast tumor-bearing mice after a single *i.v* dose administration.

### 5.1 Approaches for manufacturing targeted PFC NEs

**Figure 9:**
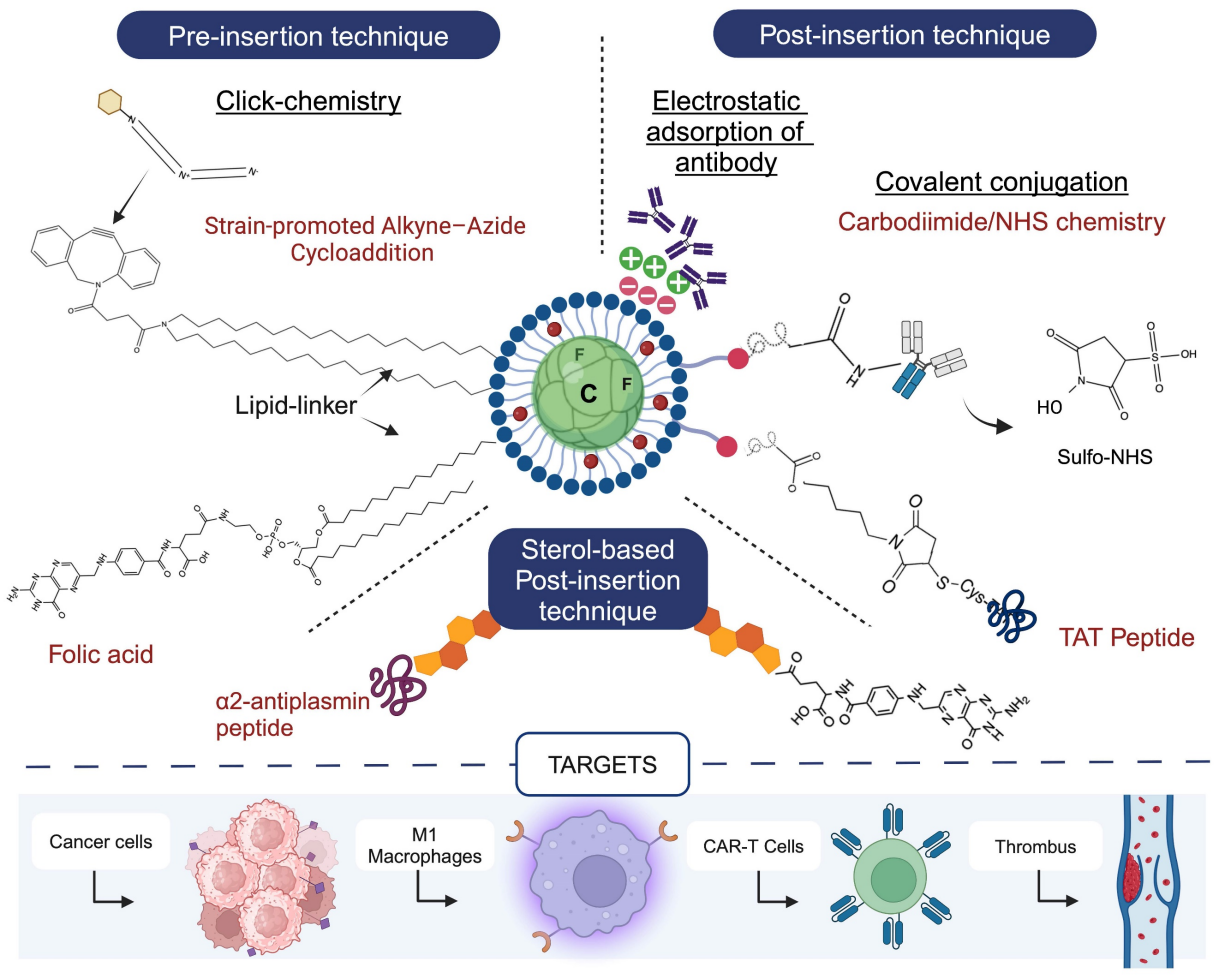
Summary of surface-functionalized PFC NEs used for drug delivery applications. Created with Biorender.com. All the chemical structures were drawn using ChemDraw 21.0.0.

The successful development of targeted PFC NEs for drug delivery applications is dependent on the choice of manufacturing strategy. Researchers must analyze the type of ligand (biomolecules or synthetic compounds) and the physicochemical properties of the selected ligand (*e.g.,* pH and heat sensitivity) before deciding at which step the selected ligand will be introduced in the manufacturing process. There are three general manufacturing strategies for developing surface-functionalized PFC NEs for active targeting **(Figure 9)**. The first strategy involves the covalent conjugation of the targeting ligand to a lipid tail anchor, such as dipalmitoyl phosphatidylethanolamine **(DPPE)** 38 or dioctadecylamine 114. Subsequently, a lower concentration (0.05-5 mol%) of the targeting ligand is mixed with surfactants and PFC. The formed coarse emulsions are then subjected to high-pressure homogenization techniques (pre-insertion technique, *approach 1*). The pre-insertion technique is limited to robust synthetic ligands that are not sensitive to high sheer stress or localized heat. One example of such a ligand is folic acid 38, 55, which binds with high affinity (K_d_∼ 0.1-1 nM) to tissue-specific folate receptors (FRs). For instance, overexpressed FR-α isoform in epithelial malignancies or upregulated FR-β isoform on proinflammatory macrophages. Yang et al. 38 manufactured FR-α targeting PFCE NE for delivering Osimertinib in a non-small cell lung cancer model. Folic acid was covalently conjugated to the headgroups of DPPE lipid tails and was added to phospholipids and PFCE oil to form a coarse emulsion prior to processing at 20,000 psi on a microfluidizer. The targeting ability of folic acid was preserved despite undergoing MF, resulting in a higher uptake of PFCE NEs within the FR-α-positive tumors.

In the second strategy, PFC NEs are first manufactured with chemically modified surfactants. The pre-formed PFC NEs can display reactive groups such as maleimide (-SH), N-hydroxysuccinimide (-NHS) esters, or carboxyl (-COOH) on the surface. In the next step, the targeting ligand is chemically conjugated on the surface of the nanodroplet (post-insertion technique, *approach 2*). This two-step process minimizes the exposure of sensitive ligands to harsh conditions and provides control over ligand conjugation. Nevertheless, careful optimization of manufacturing parameters is required to avoid the degradation of reactive groups or chemical handles. Macromolecules like antibodies 35, 36, 115 or peptides 111 are surface-functionalized on PFC NEs through *approach 2*. Lee YH *et al.* 35 immobilized an anti-human epidermal growth factor receptor 2 **(HER2)** monoclonal antibody on the surface of the doxorubicin-loaded PFC NE. First, PFOB was emulsified with carboxyl-terminated pluronic F-68 block copolymer (CT-F68), which allowed the polycationic PEI polymer to react with the negative surface charge of carboxyl moieties. Later, the HER2 antibody was immobilized on the surface through ionic adsorption. Surface conjugation with HER2 resulted in a three-fold higher uptake in the HER2+ breast cancer cell line. The same group also functionalized PFOB NE with an anti-human epidermal growth factor receptor **(anti-EGFR)** antibody and encapsulated two payloads (ICG dye and Mitomycin C) to specifically target overexpressed EGFR in metastatic bladder cancer 36. In another example, Bae *et al.* 115 proposed covalent coupling based on the carbodiimide/NHS chemistry that offers higher stability due to covalent linking between the primary amines present in the anti-HER2 antibody and the PEGylated phospholipid NSH-ester based surfactant. Both groups successfully demonstrated preservation of bioactivity following conjugation of the antibody to the surface of the PFC NE droplet. The spatial orientation or density of the antibody molecules on the PFOB NE surface are the critical parameters that directly influence the antigen-binding capacity. To prevent random immobilization of antibodies on the surface of nanoemulsions, site-selective antibody conjugation approaches like maleimide chemistry have been studied. Such chemistries can ensure “end-on” antibody immobilization on PFC NEs scaffolds. For instance, Murphy *et al.* 43 used maleimide-conjugated lipids to attach as1411 aptamer via the thiol groups at the 3' end.

The third strategy is based on using cholesterol anchors, where the cholesterol concentration is several-fold higher than the selected ligand. The developed cholesterol-ligand conjugates are inserted spontaneously in the phospholipid surfactant monolayer of pre-formed PFC NE (sterol-based post-insertion technique (SPIT), *approach 3*). The popularity of the SPIT is driven by its advantages, including mild processing conditions for ligand insertion and the feasibility of using a small volume of PFC NE. This can allow for routine ligand screening experiments. Temme *et al.* 116 developed first-in-class α2-antiplasmin peptide **(α2^AP^)**-labeled thrombin-inhibiting PFCE NEs using SPIT. The α2^AP^-PFCE NE specifically targeted factor XIIIa (FXIIIa) in developing thrombi (diameter <1 mm). SPIT provides flexibility to researchers for manufacturing PFC NEs using high sheer techniques, as the spontaneous insertion of cholesterol-ligand conjugates into the surfactant layer occurs under mild post-insertion conditions, such as shaking at room temperature.

## 6. Conclusions and Future Outlook

The first step in the preclinical development of PFC NEs for drug delivery applications involves screening and selection of formulation components. The presented review highlighted different studies that showed PFC NEs can be developed as smart nanomedicines by carefully selecting formulation components. For example, through rational selection of PFCs, formulators can design PFC NEs that can respond to exogenous stimuli, like ultrasound energy or pH alterations, to enable site-specific drug delivery. PFC NEs can also be designed to co-deliver therapeutic and diagnostic payloads or to facilitate targeted drug delivery. Based on the literature review, the interdisciplinary nature of formulation science for PFC NEs was evident. Material and formulation scientists are actively synthesizing novel polymeric surfactants for emulsifying PFC in water. However, it is important to address questions regarding the *in vivo* safety, biodistribution, and batch-to-batch consistency of these synthetic polymers. This review highlighted the potential limitations in the published academic literature on PFC NEs. It is recommended to provide detailed information on the selected critical processing parameters. Such collective efforts would help to integrate scientific findings in the field and facilitate a smooth transition of technology from academia to industry. Additionally, with advances in artificial intelligence and the development of machine learning algorithms to predict formulation parameters of nanomedicines, comprehensive data must be published for developing reliable mathematical models in the future.

Research efforts are also required to develop analytical methods or devices that would enable industries to perform in-line monitoring of the physicochemical characteristics of PFC NEs during manufacturing. Conventional DLS has several limitations, such as it relies on end-point size measurements and cannot be applied to turbid samples or in liquid flow conditions. Using advanced techniques like spatially resolved DLS (SR-DLS), which is based on the Fourier Domain Low Coherence Interferometry, can allow fast (in sec) size measurements of undiluted samples without interrupting the liquid flow. Recently, Bardsley *et al.* 117 designed optical sensors measuring turbidity that can be placed in the fluid path during the manufacturing of PFC NEs. These sensors have an ability to inform on the particle size changes in real time. The information obtained from such sensors can provide immediate feedback and guide manufacturers to optimize processing parameters like duration of homogenization in order to ensure product consistency and process reproducibility, especially during scale-up of PFC NEs.

It is also important that the formulation design of drug-delivering PFC NEs should not be overcomplicated. While this may result in publication in scientific journals, the clinical translational potential of the formulation in the future would become highly questionable. Perhaps a lesson to be learned from the discrepancy between the number of published articles on nanoparticles and commercially available nanomedicines is that during the early stages of development, researchers should think from a translational perspective when designing nanomedicines like PFC NEs. Although the progress of developing PFC NEs for drug delivery has been more like a marathon than a sprint, the field has made tremendous progress in recent years, expanding the applicability of PFC NEs to not only deliver small molecules but also biologics.

## Figures and Tables

**Table 1:** Summary of different therapeutic payloads that are delivered through PFC NEs, indication, PFC type, targeting mechanism, manufacturing method, Z-average size analyzed by dynamic light scattering (DLS), and route of delivery.

	Drugs	Indication	Type of PFC	Targeting mechanism	Manufacturing conditions	Size (nm)	Route	Ref.
Small molecules	Rapamycin	Muscular dystrophy	PFOB	-	MF, M110S- 4 minutes 20,000 psi	~185	Intra-venous	16
	Celecoxib^*^	Neuro-inflammation	PFCE	-	MF on ice M110S- 6 passes 17,500 psi	~140	Intra-venous	17
	Osimertinib (EGFR-TK1)	Non-small cell lung cancer	PFCE	Folic acid	M110P- 4 minutes 20,000 psi	100.9 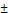 8.0	Intra-venous	38
	Resveratrol^*^	Anti-inflammatory	PFPE	-	MF on ice M110S- 6 passes 17,500 psi	155.1 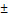 3.1	-	39
	Paclitaxel	Ovarian cancer	PFP	US-TDD	UE on ice Power-500 W	~250	Intra-venous	40
	Sn2 lipase labile-Fumagillin prodrug	Angiogenesis	PFOB	αvβ3-integrin	MF, M110S- 4 minutes 20,000 psi	~230	Intra-venous	42
	Thymoquinone	Cancer	PFP	as1411 aptamer	UE on ice Amplitude-60% Pulse mode	~195	-	43
	Rifampicin^#^	Antimicrobial	PFOB	-	UE on ice 5-10 minutes	238.6 ± 7.51	Intra-venous	37
	Doxorubicin^#^	Breast Cancer	PFOB	Anti-HER2	UE on ice	~340	-	35
	Isoflurane	General Anesthesia	PFTBA		MF on ice, M110Y- 8 minutes 8-10,000 psi	~125	Intra-venous	41
siRNA	siSTAT3	Idiopathic Pulmonary Fibrosis	PFOB	CXCR4	UE on ice Amplitude-30% Pulse mode	175 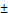 2.0	Intra-tracheal	18
	siNGF	Pancreatic cancer	PFOB	CXCR4	UE on ice Power-200 W Amplitude-80% Pulse mode	~147	Intra-peritoneal	44
	siSTAT3	Lung metastatic osteosarcoma	PFOB	CXCR4	UE on ice Amplitude-30% Pulse mode	~170	Intra-tracheal	45
Proteins	Model protein-GFP	-	PFH/ PFP	US-TDD and Folic acid	Vigorous vortexing	~300	Intra-vascular	19
	Pigment epithelium-derived factor	Acute myocardial injury	PFOB	-	Miniextruder	140 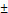 4.7	Intra-tracheal	46
Peptides and enzymes	PPACK	Atherosclerosis	PFOB	-	MF, M110S- 4 minutes 20,000 psi	160.5 0 ± 2.6	Intra-venous	48
	Melittin	Cancer	PFOB	αvβ3-integrin		~7	Intra-venous	49
	Lactate oxidase	Breast Cancer	PFTBA	PD-1-expressing cell membrane	UE 260 W 8 minutes	133.1	Intra-venous	50
pDNA	Model oligonucleotide-eGFP	-	PFOB	-	UE on ice Power-125 W Amplitude-35% Continuous mode	~170	-	51

**Abbreviations:** EGFR-TKI: Epidermal Growth Factor Receptor-Tyrosine Kinase Inhibitor, siSTAT3: Signal transducer and activator of transcription 3, siNGF: Nerve growth factor, PPACK: Phe-Pro-Arg-Chloromethylketone, US-TDD: Ultrasound-Targeted drug delivery, CXCR4: C-X-C chemokine receptor type 4, as1411: G-quadruplex aptamer, MF: Microfluidization, UE: Ultrasound Emulsification. * Triphasic PFC NEs, ^#^ W_1_/PFC/W_2_ double nanoemulsion.
